# Preface

**DOI:** 10.1007/s13280-018-1029-8

**Published:** 2018-03-07

**Authors:** Kirsten Hastrup, Bjarne Grønnow, Anders Mosbech

**Affiliations:** 1 Department of Anthropology, University of Copenhagen, Øster Farimagsgade 5, 1353 Copenhagen K, Denmark; 2 The National Museum of Denmark, Frederiksholms Kanal 12, 1220 Copenhagen K, Denmark; 3 Arctic Research Centre, Department of Bioscience, Aarhus University, Frederiksborgvej 399, 4000 Roskilde, Denmark

**Fig. 1:**
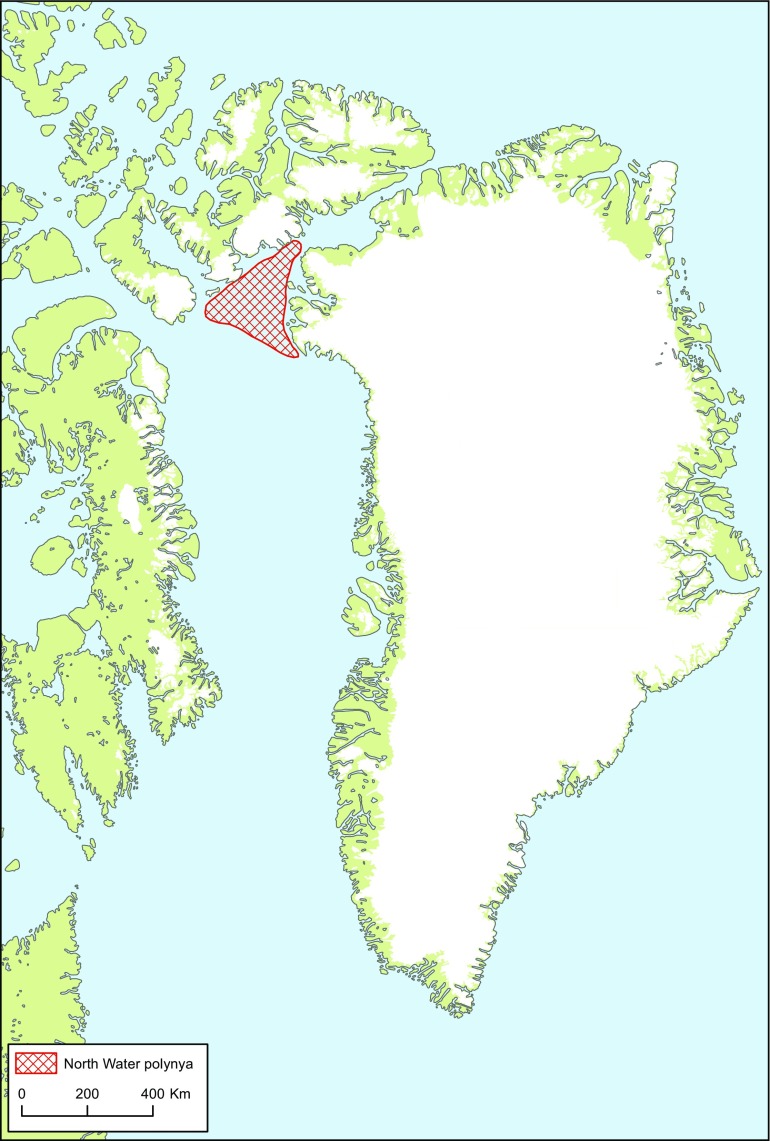
Map showing the geographical position of the North Water (marked in red) between Ellesmere Island in Northeast Canada and Northwest Greenland (~ 76° N to 79° N and 70° W to 80° W)

**Fig. 2:**
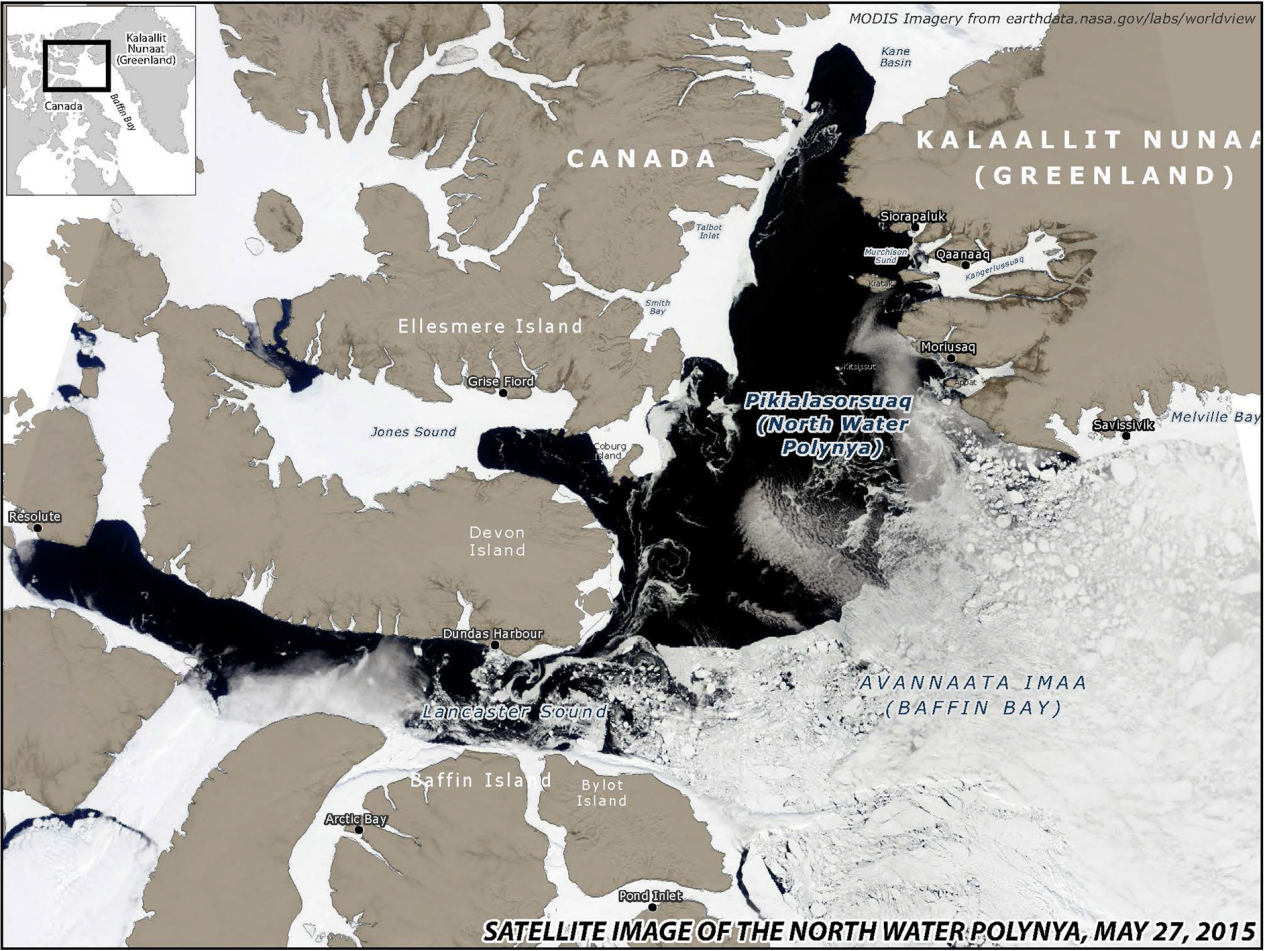
This satellite image clearly shows the extent of the open water in May. It is still surrounded by sea ice, blocking the passage southwards

This Special Issue of *Ambio* is a product of an interdisciplinary, collaborative research project centring on the North Water (NOW). The North Water is a recurrent polynya at the top of Baffin Bay, between High Arctic Canada and Northwest Greenland (Figs. 1 and 2). The research project, known as the NOW project, aimed at uncovering the dynamic relations between living resources and hunting societies in the Thule Region in a long-term perspective. The group of researchers incorporated archaeologists, biologists, and anthropologists each having their individual field of analytical expertise. Some have taken part in the joint project throughout the years, while others have been engaged for a field season or two. A sense of collectivity across disciplines has been nurtured through joint fieldwork ventures in the region and through regular interdisciplinary workshops. In the present collection of articles, we have deliberately attempted at integrating contributions from the different disciplines in all chapters, if with shifting emphases.

In all of the articles below, the North Water (NOW) is the central figure. It is created and maintained by a complex and delicate balance between topographical, meteorological, and hydrographical conditions, allowing for a large production of phytoplankton at the basic level of the marine food chain. The NOW is extremely productive in terms of primary biomass and provides food and space for an abundance of living resources like seal, walrus, narwhal, polar bear as well as various sea bird species.

The living resources in the North Water have allowed human life in an otherwise barren High Arctic landscape. Throughout 4500 years, the polynya has attracted people, who migrated from Arctic Canada into Northwest Greenland, and the rich animal life continues to be fundamental to the maritime hunting of the Inughuit in the Thule Region (also known as Avanersuaq, meaning ‘the big North’ in Greenlandic). From the early nineteenth century onwards, Europeans, who first named this polynya the *North Water*, delighted in its riches of whale and other marine mammals. Later, Knud Rasmussen’s famous Thule trading station, established in 1910 and from which the entire region was named, benefited from a variety of resources from the NOW, creating new commercial possibilities.

While constituting an oasis of open water, NOW is circumscribed by the sea ice during a substantial part of the year. Both human and other life forms in and around the polynya depend as much on the presence of the ice and the ice edge, as on the open water as such. Since the 1990s, the extent and volume of the sea ice in the Arctic have declined drastically in a possibly irreversible process. This potentially has profound impacts on both animal populations and social communities in the region, facing severe instabilities in their resource base. The NOW project has responded to the urgent need to understand the dynamic ecology of harvested marine mammal and bird populations as well as human subsistence strategies in the polynya area.

In this Special Issue, this dynamic ecology has been studied in both a contemporary and in a long-term historical and prehistoric perspective on the human–animal relation. Archaeologists have re-interpreted human traces on land, and palaeo-ecologists have investigated sediment cores and been able to trace salient animal arrivals in the region. The time depth allows for both new primary knowledge and for establishing a new baseline for contemporary comparisons. Such comparisons have been possible through anthropological and biological analyses of the current situation, including analyses of contaminants hailing from far beyond the region that affect both animals and people living from hunting. The last point goes to say that although the NOW project is localised it addresses increasingly pressing global concerns. As it is argued in one of the contributions, the notion of a closed ecosystem is increasingly challenged.

There seems to be no ancient, local name for the North Water—itself pointing directly towards the discovery of this polynya by people from more southerly regions. Yet, recently the term Pikialasorsuaq (‘The great upwelling’) has gained currency, and a Pikialasorsuaq Commission has been constituted with the aim at protecting the habitat through proper management, in the interest of animal as well as human life in the region. In this Special Issue, we have mainly referred to the North Water by its English name, by which it is mostly known to the scientific community. Yet, the Greenlandic name also figures in articles with a contemporary focus. A similar observation can be made for place names more generally; the authors may refer to Avanersuaq or the Thule Region for the same geographical space, as it appears most appropriate in the given context. However, where possible both the Greenlandic and the English place-names are mentioned the first time it is used in each paper. All of this goes to say that we have largely left it to the authors’ discretion when it comes to the use of maps and place-names in relation to a particular focus. Each article has its own tenet and core argument, and as editors we have not insisted on a uniformity of language.

A parallel issue relates to the population of the region. Archaeologically speaking, they descend from the Thule Inuit, immigrating from North America c. 1200 AD and gradually (re-) populating Greenland over the following centuries. After the establishment of the Thule Station in 1910, the inhabitants of the region were often referred to as the Thule people; the prehistoric Thule Inuit were equally named from this station, where some of their emblematic utensils were first found in an ancient midden excavated in 1916. Today the inhabitants name themselves Inughuit, if they must have a name, but they also readily accept the old name. The challenge is not to take any name as a given, or as an indication of uniformity among the c. 750 inhabitants in the region, some of whom hail from other places in Greenland or Denmark. The name Inughuit has some currency when it comes to local politics vis-à-vis the rest of Greenland, but in practice people see themselves also as Greenlanders. Again, a rigid naming policy could have thwarted the argument of particular articles.

We hope that the results published here may contribute to a more comprehensive understanding of the dynamics of a particular High Arctic ecology including its human inhabitants, and that it may eventually contribute to facilitate informed decision-making in times of drastic political, environmental, and social changes in the Arctic and beyond.

